# Current-reported outcome domains in studies of adults with a focus on the treatment of tinnitus: protocol for a systematic review

**DOI:** 10.1136/bmjopen-2015-009091

**Published:** 2015-11-11

**Authors:** Deborah A Hall, Agnieszka J Szczepek, Veronica Kennedy, Haúla Haider

**Affiliations:** 1 National Institute for Health Research (NIHR) Nottingham Hearing Biomedical Research Unit, Nottingham, UK; 2 Otology and Hearing Group, Division of Clinical Neuroscience, School of Medicine, University of Nottingham, Nottingham, UK; 3 Department of Otolaryngology, Charité-Universitätsmedizin Berlin, Berlin, Germany; 4 Department of Audiovestibular Medicine, Bolton NHS Foundation Trust, Halliwell Health and Children's Centre, Bolton, UK; 5 ENT Department of Hospital Cuf Infante Santo—Nova Medical School, Lisbon, Portugal

## Abstract

**Introduction:**

In Europe alone, over 70 million people experience tinnitus. Despite its considerable socioeconomic relevance, progress in developing successful treatments has been limited. Clinical effectiveness is judged according to change in primary outcome measures, but because tinnitus is a subjective condition, the definition of outcomes is challenging and it remains unclear which distinct aspects of tinnitus (ie, ‘domains’) are most relevant for assessment. The development of a minimum outcome reporting standard would go a long way towards addressing these problems. In 2006, a consensus meeting recommended using 1 of 4 questionnaires for tinnitus severity as an outcome in clinical trials, in part because of availability in different language translations. Our initiative takes an approach motivated by clinimetrics, first by determining what to measure before seeking to determine how to measure it. Agreeing on the domains that contribute to tinnitus severity (ie, ‘*what’*) is the first step towards achieving a minimum outcome reporting standard for tinnitus that has been reached via a methodologically rigorous and transparent process.

**Methods and analysis:**

Deciding what should be the core set of outcomes requires a great deal of discussion and so lends itself well to international effort. This protocol lays out the first-step methodology in defining a Core Domain Set for clinical trials of tinnitus by establishing existing knowledge and practice with respect to which outcome domains have been measured and which instruments used in recent registered and published clinical trials.

**Ethics and dissemination:**

No ethical issues are foreseen. Findings will be reported at national and international ear, nose and throat (ENT) and audiology conferences and in a peer-reviewed journal, using PRISMA (Preferred Reporting Items for Systematic reviews and Meta-analysis) guidelines.

**Trial registration number:**

The systematic review protocol is registered on PROSPERO (International Prospective Register of Systematic Reviews): CRD42015017525.

**Strengths and limitations of this study**

- The protocol addresses important questions relevant to weaknesses in the design of controlled trials assessing clinical efficacy of treatments for tinnitus.
- Review contributors span a number of European countries, bringing an international point of view.
- Clearly established purpose and well-defined methods for data collection and synthesis.
- Review will generate independent evidence from professional stakeholder groups.
- The anticipated heterogeneity across studies and low-quality reporting may pose challenges for the data synthesis.

## Introduction

Tinnitus is a common symptom arising from a range of otological and non-otological conditions, affecting 10–20% of the adult population, and rising with age.^1^ Tinnitus is described as a ringing, buzzing or hissing sound heard in the ears or head. Most forms of tinnitus have no physically identifiable source of the sound and so they cannot be objectively measured. This is the condition known as subjective tinnitus. Throughout this protocol, we will refer simply to ‘tinnitus’, without limiting our question to one subtype or another. The tinnitus experience can be characterised by the perceptual properties of the sound percept, namely its pitch, timbre and loudness. Yet these perceptual attributes relate poorly to the degree of self-reported functional impact on daily life.^2^ Instead, disability arising from tinnitus can be described as a multidimensional concept relating to impairments, activity limitations and participation restrictions that people with tinnitus may experience, not forgetting the environmental factors which affect these experiences.^3–5^ In this sense, tinnitus is construed within the biopsychosocial model of disability proposed by the WHO.

General practitioners and ear, nose and throat (ENT) consultants play an important role in the clinical assessment and management of tinnitus, but across countries patients may be referred to a range of other clinical stakeholders including neurologists, radiologists, audiologists, psychologists and psychiatrists.^6^ Given the interdisciplinary approach, it is unsurprising that there are a number of different strategies for managing tinnitus in adults. These include, but are not restricted to, psychoeducation and advice, provision of hearing aids, sound generators or other acoustic devices, physical therapy, psychological counselling and complementary therapies.^7^ ^8^ There are no current drugs that are licensed for alleviating tinnitus, but for managing some of the comorbid symptoms such as depression and insomnia, pharmacological approaches are prescribed.^8^ ^9^ Generally speaking, choice of clinical intervention is a multifactorial decision based on the experience of the treating clinicians, their assessment of patient needs and the broader healthcare context.^6^ Few professional bodies work to specific clinical practice guidelines or protocols for tinnitus.^10^

It is good clinical practice that treatment decisions between healthcare professionals and their patients should be supported by informed conversations about the evidence for treatment efficacy. The best-quality evidence for treatment effectiveness is drawn from randomised controlled trials and systematic reviews that synthesise the findings from those studies.^11^ Where it is not possible to design a trial that has random allocation to intervention groups, certain types of controlled trials may also be considered.^12^ Of the numerous systematic reviews of tinnitus treatments that have been published over recent years, many of them reach the conclusion that few high-quality trials are available for inclusion and for those trials that are included, a range of different outcome measures preclude the pooling of findings.^9^ ^12^ ^13^ There is a growing recognition that consistency between academic centres and clinics engaged in tinnitus research in the way that treatment outcomes are measured would facilitate more meaningful evaluation and comparison of trial findings.^3^ ^14^ ^15^ There is also agreement that the heterogeneous nature of tinnitus symptoms and impact can also make clinical research and outcome measurement difficult. Moreover, there is increasing evidence for cooperative working to address these challenges. The creation of a comprehensive and a brief core set for hearing loss^16^ is an example of the successful product of international collaboration in hearing.

In 2006, a meeting in Regensburg of 29 tinnitus professionals sought consensus for patient with tinnitus assessment and treatment outcome measurement. They recommended using one of four standard questionnaires (Tinnitus Handicap Inventory, Tinnitus Handicap Questionnaire, Tinnitus Reactions Questionnaire or Tinnitus Questionnaire) as an outcome instrument in therapeutic trials, although most were developed for intake assessment. Part of the rationale for this choice was motivated by pragmatism (eg, availability in different language translations). These are all multi-item questionnaires that purport to measure tinnitus severity, but do so using different questions, rating scales and subscales. Moreover, the design of the four recommended questionnaires was optimised for intake assessment of tinnitus, rather than for evaluation of treatment-related change.^3^ ^17^ Clearly, there is more work to be done. We keep our minds open that recommendations for outcome measurement do not necessarily need to be restricted to questionnaires. For example, in chronic pain, biological markers and clinician ratings of global improvement are proposed as supplemental domains for clinical trials.^18^

**Figure 1:**
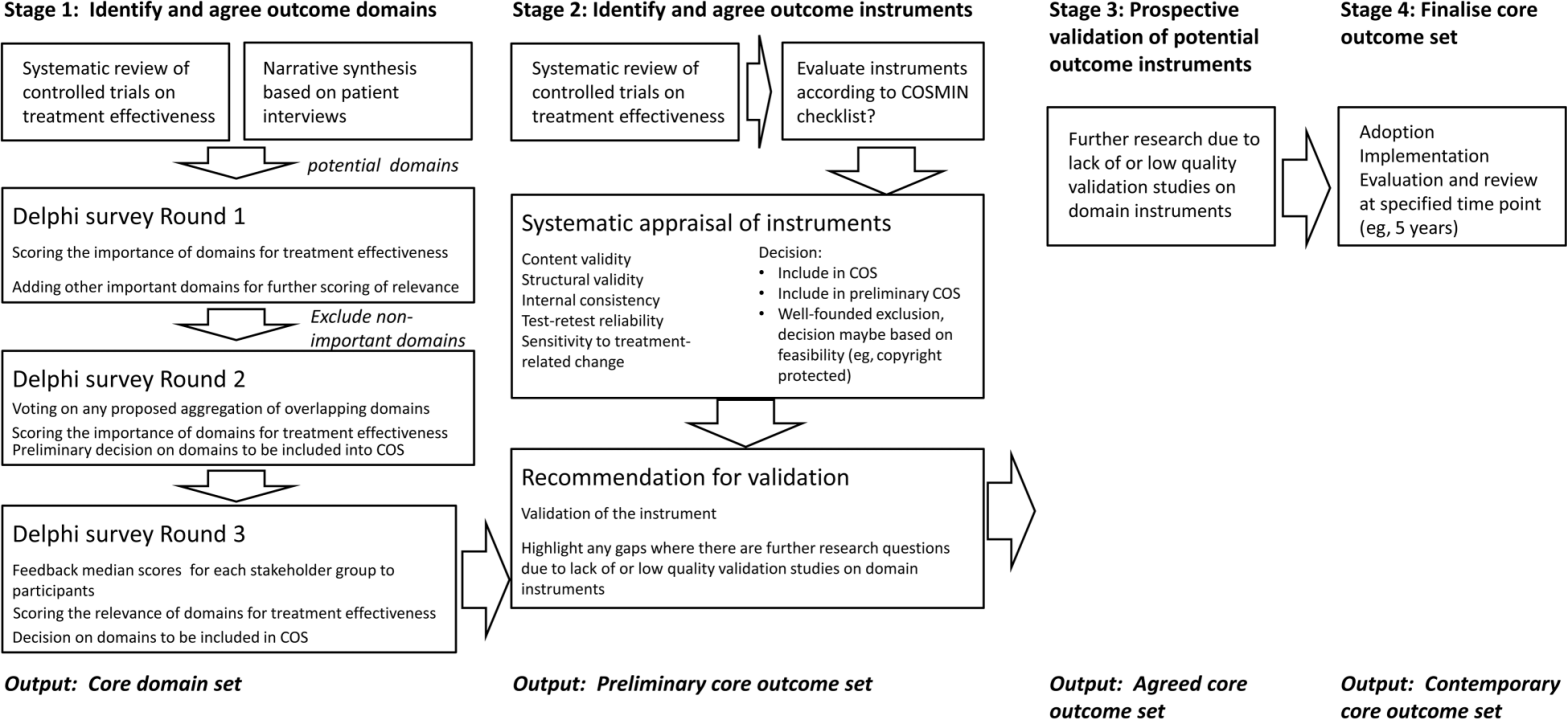
The proposed stepwise road map^15^ for developing a core outcome set. This figure is taken from a SAGE article published under the Creative Commons Attribution-NonCommercial 3.0 License that permits reproduction without further permission.^15^ COS, Core Outcome Set; COSMIN, COnsensus-based Standards for the selection of health Measurement Instruments.

Building on the 2006 consensus approach, a COST (Cooperation in Science and Technology) Action network for tinnitus (TINNET) is promoting the international coordination of nationally funded research across Europe (http://tinnet.tinnitusresearch.net/). The network has five working groups, of which Working Group 5 has the goal to establish an international standard for outcome measurements in clinical trials of tinnitus. Our activity is conducted as the COMiT (Core Outcome Measures in Tinnitus) initiative which is part of Working Group 5. In a recent article, we promoted a stepwise road map^15^ (figure 1) for developing a core outcome set. This approach is inspired by the work of the COMET (Core Outcome Measures in Effectiveness Trials) initiative.^19^ The first output will be a consensus on what outcome *domains* are essential (ie, core) to be captured in all controlled trials. A domain is defined as a distinct element (or topic) of tinnitus such as how loud or how emotionally distressing a patient may find his or her tinnitus. These core domains will be identified as being important for characterising tinnitus and will reflect the perspectives of professionals and lay people alike. This article describes a systematic review protocol to be implemented within stage 1 of that road map. It relates to current reported outcome domains in studies of adults with a focus on the treatment of tinnitus. International agreement on a core outcome domain set would drive up the quality and relevance of research by ensuring that the most relevant outcomes are consistently measured and reported in trials relating to tinnitus.

The aim of the first systematic review is to contribute to the development of a core domain set for future controlled trials on tinnitus treatment effectiveness using *quantitative* data collected from ongoing registered or published trials since the 2006 consensus meeting.^14^ This represents the professional perspective on outcome domains in clinical trials of tinnitus. The objective of the review is to conduct a worldwide search of the recent published literature of clinical trials of interventions for tinnitus, as well as those ongoing trials that are publicly registered. More specifically, this objective will answer the research question: ‘What are the current reported outcome domains in studies of adults with a focus on the treatment of tinnitus?’.

### Methods and analysis

Methods are reported according to the Preferred Reporting Items for Systematic reviews and Meta-analyses for Protocols (PRISMA-P) 2015.^20^ ^21^ Subheadings correspond to some of the items in the PRISMA-P checklist. The allocation of specific roles to named authors of the review will be made at a later date, and this information will clearly be acknowledged in any subsequent dissemination of findings.

### Eligibility criteria

We have defined our inclusion criteria according to PICOS (Participant, Intervention, Comparison, Outcome, Setting) guidelines as follows:

- *Participants*: For maximum inclusivity, we will include all human participants with the only restrictions being adults (≥18 year) who report tinnitus as one of their primary symptoms. To be confident that our review does not include trials recruiting participants younger than 18-years, any trials specifying an age range with a lower limit than this will be excluded. We will include men and women.
- *Intervention*: Again for maximum inclusivity, we will include any intervention irrespective of whether it is a clinical intervention or a novel experimental intervention. The key criterion is that the main aim of the intervention is to achieve a therapeutic benefit for people with tinnitus, not those in whom the impact on tinnitus might be of secondary benefit.
- *Comparison:* The following types of study comparisons will be eligible for inclusion: randomised controlled trials, before and after studies, non-randomised controlled trials or case–control studies and cohort studies. We will also include published meta-analysis and systematic reviews that have considered such studies. However, we will exclude articles reporting expert opinions, practice guidelines, case reports, case series, conference abstracts and book chapters due to their more limited clinical and scientific value. In accordance with this rationale, the search strategy will also exclude those trials either recruiting fewer than 20 participants with tinnitus, or having fewer than 20 at the end of follow-up. This cut-off is somewhat arbitrarily selected, but follows Needleman *et al*.^22^
- *Outcomes*: Since the research question has a focus on the treatment of tinnitus, studies will be restricted to any changes related to tinnitus as a primary outcome, irrespective of how these are measured.
- *Settings*: Any research settings will be included, notably clinical and academic sites. This is consistent with our approach to include all participants and interventions for tinnitus, including exploratory therapies recruiting non-clinical groups.

Two review eligibility characteristics are specified. First, articles will be in the English language. Second, articles must be published (in print copy, not published online first) on or after July 2006. This date is selected to follow from the first international consensus meeting for patient with tinnitus assessment and treatment outcome measurement.^14^ For registered clinical trials, we will apply the same publication date criteria so that our search will include only those studies that are first received after July 2006. However, by doing so, we acknowledge the potential limitation of this strategy which is that included study designs may have been approved and open for recruitment before the Regensburg consensus meeting in July 2006.

### Information sources

Intended information sources are the following electronic research databases: PubMed (National Center for Biotechnology Information), EMBASE (Ovid) and Cumulative Index to Nursing and Allied Health Literature (CINAHL, EBSCO), and trial registers: ClinicalTrials.gov, the International Standard Randomised Controlled Trial Number registry (ISRCTN, BioMed Central), International Clinical Trials Registry Platform (ICTRP, WHO) and the Cochrane Database of Systematic Reviews (CDSR). We will also search the Cochrane Central Register of Controlled Trials (CENTRAL) which contains a highly concentrated source of reports of randomised controlled trials. With the exception of trial registers, we will not use databases that are specialised in grey literature.

Additional information will be identified by a manual search of the registered clinical trials to identify any additional registers of the trial and to identify any published protocols or study findings that are indexed to a particular trial by its unique study identifier, but that have not already been identified by the electronic research database search. Furthermore, we will manually search all the systematic review articles found in order to seek any further trials for inclusion. This will be restricted to those studies that met eligibility for inclusion in the corresponding review publication.

Following title exclusion, the next phase will assess eligibility based on the full text, accessing hard-to-reach records via the British Library where necessary. Contact with study authors will be permitted.

The database search was conducted soon after registration in PROSPERO (12–13 March 2015) by authors DAH and AJS. The manual search and personal author contact will be ongoing up to the end of the data collection phase.

### Search strategy

The systematic review attempts to collate all relevant evidence that fits prespecified eligibility criteria to answer our specific research question. The electronic database search strategy will use a combination of Medical Subject Headings (MeSH) and relevant text words wherever possible. The search terms for PubMed, EMBASE and CINAHL are: (tinnitus) AND (stud* OR clinical trial* OR therap* OR treatment* OR intervention). For example, the search strategy for Embase will be: (((((((((“tinnitus” and “stud*)” or “Tinnitus)” and “clinical trial*)” or “Tinnitus)” and “Therap*)” or “Tinnitus)” and ‘Treatment*)” or “Tinnitus)’ and “Intervention*)’.mp. (mp=title, abstract, subject headings, heading word, drug trade name, original title, device manufacturer, drug manufacturer, device trade name, keyword). Limit to (english language and (embase or medline) and yr=“2006 -Current” and (article or “review”) and (adult <18 to 64 years> or aged <65+years>)). This will be adapted to the syntax and subject headings of the other databases. The authors do not have access to a health information specialist with database searching skills, and so the most experienced researchers will conduct the search.

### Data management

DAH (author) will be responsible for data management and will have editorial rights. All identified records will be saved into an Excel master file where records can be tracked through the screening and data collection process. A simple system of record annotation will be implemented to capture reasons for exclusion. Included records will be allocated a study ID code to link each record in the master file with its corresponding full text and data collection sheet.

### Selection process

Endnote will be used to remove duplicate records from the PubMed, EMBASE and CINAHL searches. One of the review authors will then manually screen all records for duplicates using author names, study title and trial registration number. A two-step process will then be implemented to decide eligibility according to the inclusion and exclusion criteria: first by reading the title, and second by reading the full text. The full text will be obtained for all potentially relevant records appearing to meet the inclusion criteria or for which there is insufficient information in the title to make a clear decision. Screening steps will be carried out by a pool of three authors (DAH, AJS and HH), while data collection will be conducted by a pool of 20 project team members. We will adopt the principle that two project team members will always perform each key step independently for every record (ie, title screening, full text screening and data collection). Discrepancies will be resolved by DAH or one other designated project team member (in cases when DAH is the screener or data extractor) acting as an arbitrator. Inter-rater agreement will not be calculated.

We will piece together data from multiple reports of the same study by manually screening all included records using author names, study title and trial registration number.

### Data collection process

Data collection will be guided by an electronic form (Excel spreadsheet) that will also be used to collate all responses. To ensure consistency across reviewers, a full set of guidance notes will be produced for the data collection procedure and calibration exercises will be conducted with new members of the review team prior to any individual contribution to this review. The sheet and the guidance notes will be developed and revised through two iterations of piloting across several review authors. Data collection will be conducted independently and in duplicate (two people) for every included record. For any studies where insufficient detail is given to determine eligibility (eg, minimum age of enrolment or sample size), we will contact the corresponding author by email (without a reminder) to seek clarification.

Many of the review authors will not have English as their native language and so DAH will sample a subset (<5%) of data collection sheets for each author performing this step, again in order to confirm consistency of approach.

### Data items

The data collection sheet will include a list of fields relating to trial design and methodology. These are given in box 1. If any information is not reported, then ‘not stated’ will be recorded in the corresponding field. For those records in which several pieces of information are consolidated into a single record, we will seek to detect any modifications to the methods and any selective reporting in the completed reports of the study findings.

Box 1 **Data items for the systematic review of trials on treatment effectiveness**

Descriptive checklist

- Study ID code
- Study title
- Name and contact details of corresponding author
- Country where study is conducted
- Date of publication (month, year)
- Date of study start (month, year)
- Study design
  Randomised controlled trials
  Before and after studies
  Non-randomised controlled trials or case–control studies
  Cohort study
  Other
- Aim of study
- Type of intervention (all arms)
- Duration of intervention
- Sample size calculation (if ‘yes’, give details)
- Sample size
- Age range
- Inclusion criteria relating to tinnitus
  Duration
  Intermittent or constant
  Pulsatile or non-pulsatile
  Severity
  Any other subtypes
- Exclusion criteria relating to tinnitus
- Inclusion criteria relating to other health-related comorbidities
- Exclusion criteria relating to other health-related comorbidities
- Primary outcome domain(s)
- Primary outcome instrument(s)
- Time frame: primary outcome(s)
- Secondary outcome domain(s)
- Secondary outcome instrument(s)
- Time frame: secondary outcome(s)
- Description of any modifications to the methods, particularly any information that might be interpreted as reporting bias
- Notes (this optional field will be used to record any further comments that may be deemed informative. In particular, whether a clinical interpretation of outcome instrument scores was defined and whether adverse events or side effects were reported as study findings)

### Outcomes and prioritisation

The main aim of the review focuses on changes related to tinnitus as a primary outcome and so the data relating to primary outcome domains and instruments will be the priority for data synthesis and reporting of findings. These outcomes link directly to the first two bullet points described in the section on Data synthesis.

### Risk of bias in individual studies

Bias is typically considered to be a systematic error that can lead to an overestimation or underestimation of the true intervention effect. Given that the primary objective of this systematic review concerns methodology, not findings, we have limited plans to assess risk of bias of individual studies. However, the data collection for consolidated records will enable summary statistics and a narrative synthesis about selective outcome reporting (reporting bias) either where original descriptions of outcomes as ‘primary’ or ‘secondary’ may have been altered retrospectively in the light of the findings, or where a subset of the original outcomes may have been selected, on the basis of the results, for inclusion in the publication of trial findings.

The potential for observer bias during the data collection process will be reduced by avoiding any instance where an individual will extract data relating to one of their own trials.

### Data synthesis

The main purpose of this systematic review is to create new knowledge around the outcome domains (and instruments) that are being used in clinical trials of tinnitus by pooling together and critically evaluating these data. The identified domains and instruments will be tabulated in such a way as to illustrate five patterns in the data using all included records. The first two bullet points address the primary purpose of the review, whereas the subsequent three bullet points relate to secondary review aims.

- *Patterns across primary outcome domains* to determine the proportion of records in which it is specified, and for those that do specify a domain what that domain is. The same analysis will be conducted separately across secondary outcomes.
- *Patterns across primary outcome instruments* to determine the proportion of records in which it is specified, and for those that do specify an instrument what that instrument is and what the timing of the primary end point is for evaluating treatment efficacy. The same analysis will be conducted separately across secondary outcomes.
- *Patterns across continents* to determine whether there are geographical preferences for using one primary outcome instrument over another.
- *Patterns across years* to determine changes over time in the uptake of outcome instruments as a primary outcome, especially the recent Tinnitus Functional Index.^23^
- *Patterns across interventions* to determine whether particular classes of intervention (eg, neurophysiological, audiological, psychological, etc) favour using one primary outcome instrument over another.

Four assessments of the quality of defining and reporting outcomes are planned:

- The first will consider whether the authors’ specification of each primary outcome domain is adequate. For example, ‘tinnitus severity’ is not a domain, yet it is commonly presented as a symptom or an outcome.
- The second will consider the degree to which each primary outcome domain is appropriate and consistent with the authors’ choice of primary outcome instrument. For example, ‘tinnitus loudness’ might be measured using a Visual Analogue Scale which should be specified for loudness.
- The third will examine the extent to which the trial design is informed by a sample size calculation based on previous data for the primary outcome instrument. This is informative because published information about the treatment-related sensitivity of particular instruments, necessary to determine the statistical power of the study, is often lacking.^3^ ^17^
- The fourth will consider how many *primary* outcomes are measured in each study. To avoid outcome reporting bias, it is preferable that one outcome measure and end point is specified,^19^ but this is not always the case.

Table 1 provides an overview of the quality items to be synthesised and reported. In addition to these planned syntheses, the data collection for consolidated records will enable summary statistics and a narrative synthesis about selective outcome reporting (reporting bias).

**Table 1:** Quality items for the systematic review of trials on treatment effectiveness

Quality checklist	Count of records			
Adequate specification of primary outcome domains	*Proportion adequately specified in each record*			
	76–100% (n= )	51–75% (n= )	<50% (n= )	not stated (n= )
Appropriate mapping between primary domain and instrument	*Proportion appropriately specified in each record*			
	76–100% (n= )	51–75% (n= )	<50% (n= )	not stated (n= )
Sample size calculated for primary instrument	Yes (n= )	No (n= )	not stated (n= )	
Number of primary outcomes	*Number in each record*			
	1 (n= )	2–3 (n= )	4–5 (n= )	>5 (n= )

A final exploratory analysis of subgroups will address the question “Is a particular outcome domain (or instrument) preferentially selected in trials that enrol a particular tinnitus subtype, or when tinnitus presents with a particular comorbid condition?” This final analysis will be conducted only if there are sufficient data.

## Ethics and dissemination

No ethical issues are foreseen. The findings will be reported at national and international ENT and audiology conferences and in a peer-reviewed journal using the PRISMA (http://www.prisma-statement.org/). Publication reporting will include the checklist and the flow diagram to depict the flow of information through the different phases of the systematic reviews. All data collected according to the data items will be available on request to the extent that they are not included in the published systematic review article.
